# Apoptosis Induction in Primary Human Colorectal Cancer Cell Lines and Retarded Tumor Growth in SCID Mice by Sulforaphane

**DOI:** 10.1155/2012/415231

**Published:** 2011-07-17

**Authors:** Ming-Jenn Chen, Wei-Yu Tang, Che-Wei Hsu, Ya-Ting Tsai, June-Fu Wu, Chen-Wei Lin, Ya-Min Cheng, Yi-Chiang Hsu

**Affiliations:** ^1^Division of Traumatology, Department of Surgery, Chi Mei Medical Center, Tainan, Taiwan; ^2^Graduate Institute of Medical Science, College of Health Sciences, Chang Jung Christian University, No. 396, Sec. 1, Changrong Road, Gueiren District, Tainan City 71101, Taiwan; ^3^Department of Nutrition and Health Sciences, College of Health Sciences, Chang Jung Christian University, No. 396, Sec. 1, Changrong Road, Gueiren District, Tainan City 71101, Taiwan; ^4^Department of Obstetrics and Gynecology, Institute of Clinical Medicine, College of Medicine, National Cheng Kung University, Tainan, Taiwan; ^5^Innovative Research Center of Medicine, College of Health Sciences, Chang Jung Christian University, No. 396, Sec. 1, Changrong Road, Gueiren District, Tainan City 71101, Taiwan

## Abstract

We have investigated the anticancer effects of the dietary isothiocyanate sulforaphane (SFN) on colorectal cancer (CRC), using primary cancer cells lines isolated from five Taiwanese colorectal cancer patients as the model for colorectal cancer. SFN-treated cells accumulated in metaphase (SFN 6.25 *μ*M) and subG1 (SFN 12.5 and 25 *μ*M) as determined by flow cytometry. In addition, treated cells showed nuclear apoptotic morphology that coincided with an activation of caspase-3, and loss of mitochondrial membrane potential (ΔΨm). Incubations at higher SFN doses (12.5 and 25 *μ*M) resulted in cleavage of procaspase-3 and elevated caspase-2, -3, -8, and -9 activity, suggesting that the induction of apoptosis and the sulforaphane-induced mitosis delay at the lower dose are independently regulated. Daily SFN s.c. injections (400 micromol/kg/d for 3 weeks) in severe combined immunodeficient mice with primary human CRC (CP1 to CP5) s.c. tumors resulted in a decrease of mean tumor weight by 70% compared with vehicle-treated controls. Our findings suggest that, in addition to the known effects on cancer prevention, sulforaphane may have antitumor activity in established colorectal cancer.

## 1. Introduction

Isothiocyanates (ITCs) are natural components of certain plants and vegetables that have selective biological activities and functions against carcinogenesis [1, 2], and many chemopreventive properties have been reported [3, 4]. The use of naturally occurring compounds combined with chemotherapy might enhance drug sensitivity [5, 6]. Sulforaphane (SFN), a potent cancer preventive agent, is a dietary isothiocyanate compound found as a precursor glucosinolate in cruciferous vegetables such as Brussels sprouts, cauliflower, and broccoli [7]. Interest in this agent has grown in recent years based on its putative beneficial pharmacological effects, which include antioxidant [8], anti-inflammatory [9], and antitumor properties [10, 11]. It has been discovered that SFN is also a potent scavenger of reactive oxygen species (ROS), including superoxide anions and hydroxyl radicals [12, 13]. Moreover, there have been some indications that SFN may help in the prevention and treatment of patients with oxidative damage and suppress the specific inflammatory factors [14, 15]. Many studies indicate a positive correlation between the consumption of cruciferous vegetables and the decreased incidence of some tumors including prostate [16], cervical [17], ovarian [18], lung [19], and gastrointestinal tract [20, 21]. In addition to the actions of inhibiting cell proliferation and increasing apoptosis [22], other mechanisms have also been proposed to rationalize the anticarcinogenic effect of SFN, such as the anti-inflammatory and antioxidant activities, the induction of phase-II detoxification enzymes, the inhibition of cyclooxygenase 2 (COX-2) [23], the effect on NF*κ*B transcription factors [24], the inhibition of matrix metalloproteinase (MMP), the effect on protein kinases, and others [25, 26]. 

Effects of SFN on five established human primary cell lines (CP1 to CP5) were investigated in this study. In this paper, studies have been initiated to investigate whether the SFN could contribute to the antiproliferation and apoptosis of the primary cancer cells isolated from the colorectal cancer patients. The aims are to investigate whether the antiproliferation and apoptosis activities of SFN on the primary colon cancer cells could be explained. We expect that all of these experiments could provide a scientific basis and technical support for CRC therapy.

## 2. Materials and Methods

### 2.1. Materials

Sulforaphane [1-isothiocyanato-(4*R*,S)-(methylsulfinyl)butane], DMSO (dimethyl sulfoxide), and MTT [3-(4,5-dimethylthiazol-2-yl)-2,5-diphenyltetrazolium bromide] were obtained from Sigma (St. Louis, MO). Cell culture medium (DMEM), fetal bovine serum, antibiotics, sodium pyruvate, trypsin, and phosphate-buffered saline (PBS) were purchased from Gibco, BRL (Grand Island, NY). Polyvinylidene fluoride membrane (PVDF) (Millipore) and molecular weight markers were purchased from Bio-Rad (USA). All other reagents and compounds were analytical grades.

### 2.2. Cell Culture

The five primary cell lines of colon cancer cells were derived, as a gift, from the cell bank maintained in the MedicoGenomics Research Center at KMU [27, 28]. The cells were grown at 37°C in Dulbecco's Modified Eagle Medium (GibcoBRL) supplemented with 10% (v/v) Fetal Bovine Serum (HyClone) and a combination of antibiotics (penicillin, 200 unit/mL, and streptomycin, 200 g/mL) (HyClone) under an atmosphere CO_2_/air (5%) for this series of studies.

### 2.3. Cell Proliferation Assay

The cells were seeded into 96-well culture plates at 5000 cells/well. The cells were treated with 0, 6.25, 12.5, 25, and 50 *μ*M SFN for 1 to 4 days. MTT dye (1 mg/mL) was added to each well for at least 4 hours of treatment. The reaction was stopped by the addition of DMSO, and optical density was measured at 540 nm on a multiwell plate reader. Background absorbance of the medium in the absence of cells was subtracted. All samples were assayed in triplicate, and the mean for each experiment was calculated. Results were expressed as a percentage of control, which was considered as 100%. Each assay was carried out in triplicate, and the results were expressed as the mean (±SEM).

### 2.4. Cell Cycle Analysis

The method for cell cycle analysis using propidium iodide (PI) is using the fluorescent nucleic acid dye PI to identify the proportion of cells that are in one of the three interphase stages of the cell cycle. The cells were treated with 0, 6.25, 12.5, and 25 *μ*M SFN for 24 hours. Cells were harvested and fixed in 1 mL cold 70% ethanol at least 8 hours at −20°C. DNA was stained in PI/RNaseA solution, and the cell cycle (At least 10,000 single cells) was detected by flow cytometry (FACScalibur, BD). Data was analyzed by WinMDI 2.8 free software (BD, USA).

### 2.5. Evaluation of Apoptosis

The apoptosis was assessed by the ApopNexin FITC apoptosis detection kit (Chemicon, USA). The cells were treated with 0, 6.25, 12.5, and 25 *μ*M SFN for 6 hours, and the apoptotic cells were detected by ApopNexin FITC apoptosis detection kit and flow cytometry (FACScalibur, BD) and data analyzed by WinMDI 2.8 free software (BD, USA).

### 2.6. Evaluation of Mitochondrial Membrane Potential (MMP; ΔΨm)

The cells were first seeded in 24-well plates (Orange, UK). Following the treatment with SFN for 6 hours, JC-1 (10 *μ*g/mL, Sigma, USA) was added to the culture medium, 50 *μ*L per well, and then incubated (at 37°C for 20 min) for mitochondria staining. After washing twice with a warm PBS, the cells were fixed with 2% paraformaldehyde, inspected by Flow cytometry (FACSCalibur, BD) and data analyzed by WinMDI 2.8 free software (BD, USA).

### 2.7. Western Blot Assay

A total of 30–50 *μ*g proteins were separated by SDS-PAGE (10–12% SDS-polyacrylamide gel electrophoresis) and transferred to PVDF membranes (Millipore, USA) in a tank blotter (in 25 mM Tris/0.192 M glycine, pH 8.3/20% methanol) at 30 voltage overnight. The membranes were blocked with 5% nonfat milk (in 10 mM Tris-HCl, pH 8.0/150 mM NaCl/0.05% tween-20) overnight and incubated with anti-*β*-actin (AC-15 Sigma, USA), anti-Caspase 3 (SC-7148 Santa Cruz, USA) antibody for 1.5~2 hours. The blots were washed with Tris-HCl (pH 8.0/150 mM NaCl/0.05% Tween-20) for 3 × 10 minutes and incubated with second antibody (antirabbit or antimouse immunoglobulins) (IRDye Li-COR, USA) at 1/200 dilution for 1 hour. The antigen was then visualized and analyzed by Odyssey infrared imaging system (Odyssey LI-COR, USA).

### 2.8. Caspase Activity Assay

The caspase (2-, 3-, 8-, and 9-) activity was assessed by the ApoAlert Caspase assay plates (Clontech, USA). The cells were treated with SFN of 0, 12.5, and 25 *μ*M with or without caspase-specific inhibitor for 8 hours. The caspase activity was detected by ApoAlert Caspase assay plates and inspected by the BioTek FLx800 TBI reader (Bio-Tek, USA). The plates contained the fluorogenic substrates and inhibitors specific for different caspases. These substrates were covalently linked to their respective activated caspases. The substrates were covalently linked to the fluorogenic dye 7-amino-4-methyl coumarin (AMC). Peptide-bound AMC excites in the UV range (380 nm) and emits at 460 nm. The AMC was normalized by total protein, and each assay was carried out in triplicate, and the results were expressed as the mean (±SEM).

### 2.9. Confocal Microscopy of NF-*κ*B Subunit p50 Activity

Confocal microscopy was performed as described previously. Briefly, the CRC cells (2 × 10^6^ cells) were treated with 0 and 25 *μ*M SFN for 16 hours and were fixed on coverslips. After treatment, they were incubated with rabbit antihuman p50 antibody (SC-8414 PE, Santa Cruz Biotechnology) for 30 minutes and then washed with PBS. The cells were mounted onto microscope slides using mounting medium containing DAPI.

### 2.10. DNA Fragmentation Assay

The DNA fragmentation was detected by ApoAlert DNA fragmentation assay kit (Clontech, USA). The assay is based on terminal deoxynucleotidyl transferase-(TdT-) mediated dUTP nick end labeling (TUNEL). TdT catalyzes incorporation of fluorescein-dUTP at the free 3′-hydroxyl ends of fragmented DNA. The cells were treated with SFN for 24 hours, and the fluorescein-labeled DNA was detected via flow cytometry (FACSCalibur, BD, USA) and data analyzed by WinMDI 2.8 free software (BD, USA).

### 2.11. Tumor Xenograft Animal Model

Experiments were done on male severe combined immunodeficient (SCID) mice (National Laboratory Animal Center, Taipei, Taiwan) according to the regulation of the Institutional Animal Care and Use Committee (IACUC, CJCU-98-006). CRC (CP1 to CP5) tumors for implantation were initially grown from injections of CRC cell lines (200 *μ*L of 3 × 10^6^ cells) at the s.c. abdomen site. Tumor cells were implanted at the same site into an experimental animal at the age of 6 weeks. After 21 days of tumor establishment in severe combined immunodeficient mice, at the start of the exponential tumor growth phase, animals were randomly divided into the treatment groups (*n* = 3). Each animal received i.p. injection (200 *μ*L) either PBS (vehicle control) or SFN (200 *μ*M and 400 *μ*M) daily, including weekends, for 3 weeks. Animal body weight and tumor size were measured and recorded. Results were expressed as a percentage of vehicle control, which was considered as 100%. Each assay was carried out in triplicate, and the results were expressed as the mean (±SEM).

### 2.12. Statistical Analysis

All data were reported as the means (±SEM) of at least three separate experiments. Statistical analysis was using *t*-test, with the significant differences determined at the level of *P* < 0.05.

## 3. Results

### 3.1. SFN Inhibits the Cell Survival/Proliferation of Five Primary CRC Cell Lines

**Figure 1:**
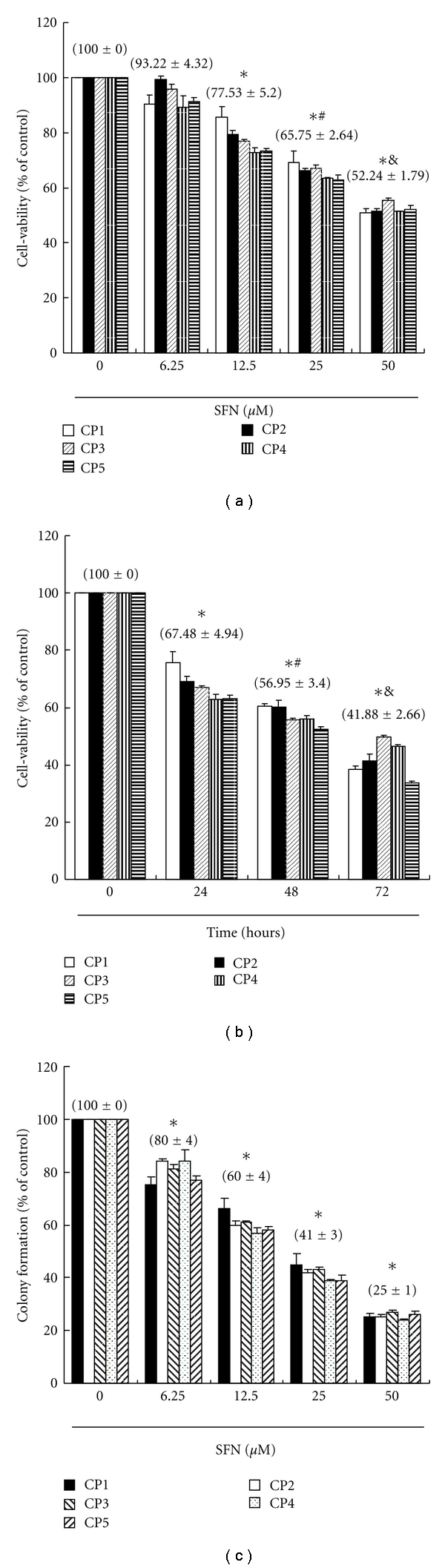
(a) Sulforaphane mediates the survival of five human primary colorectal cancer (CRC) cell lines (five groups, *n* = 6 per group) and thus inhibits their proliferation. *In vitro* study was initiated by treating each of the cell lines to the increasing doses of SFN (0, 6.25, 12.5, 25, and 50 *μ*M) for 24 hours. Statistical analysis used the *t*-test, with the significant differences determined at the level of *P < 0.05 versus control group (SFN 0 *μ*M), while the symbol on the bar denotes the difference which is statistically significant at *P* < 0.05 as compared to the 12.5 (^#^) or 25 *μ*M (^&^). (b) Reversibility of the growth inhibitory effect of SFN. The CRC cell lines were each first treated with SFN 12.5 *μ*M for 24 hours. After the treatment was terminated by washing off SFN, the cultures were reincubated for 24–72 hours to check the extent of recovery of cancer cells. The survival of these SFN-treated CRC cells was then measured by MTT method. (c) Effect of SFN on colony formation. CRC cells were treated with SFN, and number of colonies was counted. Results were expressed as a percentage of control, which was considered as 100%. All data were reported as the means (±SEM) of at least three separate experiments. Statistical analysis used the *t*-test, with the significant differences determined at the level of *P < 0.05 versus time 0 group, while the symbol on the bar denotes the difference which is statistically significant at *P* < 0.05 as compared to the time 24 (^#^) or 48 (^&^) of the recovery study.

We hypothesized that SFN could mediate the survival of primary colorectal cancer cell lines and thus inhibit their proliferation. To explore this antitumor activity of SFN against the CRC cells, an *in vitro* study was initiated by treating each of the CRC cell lines to increasing doses of SFN (0, 6.25, 12.5, 25, and 50 *μ*M) for 24 hours. The proliferation of these SFN-treated cancer cells was then measured by MTT method. The results summarized in Figure 1(a) indicate that the survival and proliferation of the primary colon cancer cells both decrease per increase of the dose of SFN added into the cell culture, which show a dose-dependent reduction (*y* = −12.3*x* + 114.65  *R* ^2^ = 0.9892). Moreover, SFN was noted to induce a morphological change in the primary colon cancer cells. A microscopic examination showed that following the exposure to SFN (25 *μ*M) for 6 to 24 hours, the primary colon cancer cells have displayed a remarkable change in their morphology and SFN induced the death of cancer cells, which formed a suspension in the medium (data not shown).

### 3.2. Growth-Inhibitory Effect of SFN Is Partially Irreversible

To study whether the growth-inhibitory effect of SFN is reversible, the primary colon cancer cells were recultivated in a fresh culture medium, after their exposure to SFN (12.5 *μ*M) for 24 hours, and the recovery of cell proliferation was then assessed for an additional 24 to 72 hours and analyzed by the MTT assay. The results in Figure 1(b) suggest that the cancer cells have substantially lost their ability to proliferate (*y* = −18.495*x* + 112.83  *R* ^2^ = 0.9393) following the SFN treatment for 24 hours. The observations could imply that the primary colorectal cancer cells have undergone an irreversible change, such as apoptosis, at least to a partial extent.

### 3.3. Sulforaphane Inhibits Colony Formation in Human CRC Cells

We next examined the effects of SFN on colony formation (a characteristic of cancer) on five CRC cell lines by soft agar assay. SFN inhibited colony formation in a dose-dependent manner (Figure 1(c)). Colonies formed by CRC cells were sensitive to SFN. These data suggest that SFN can be used as a potent chemopreventive agent for CRC therapy.

### 3.4. SFN Treatment Induces Cell Cycle G2/M Arrest and Accumulated SubG1 in CRC Cell Lines

**Figure 2:**
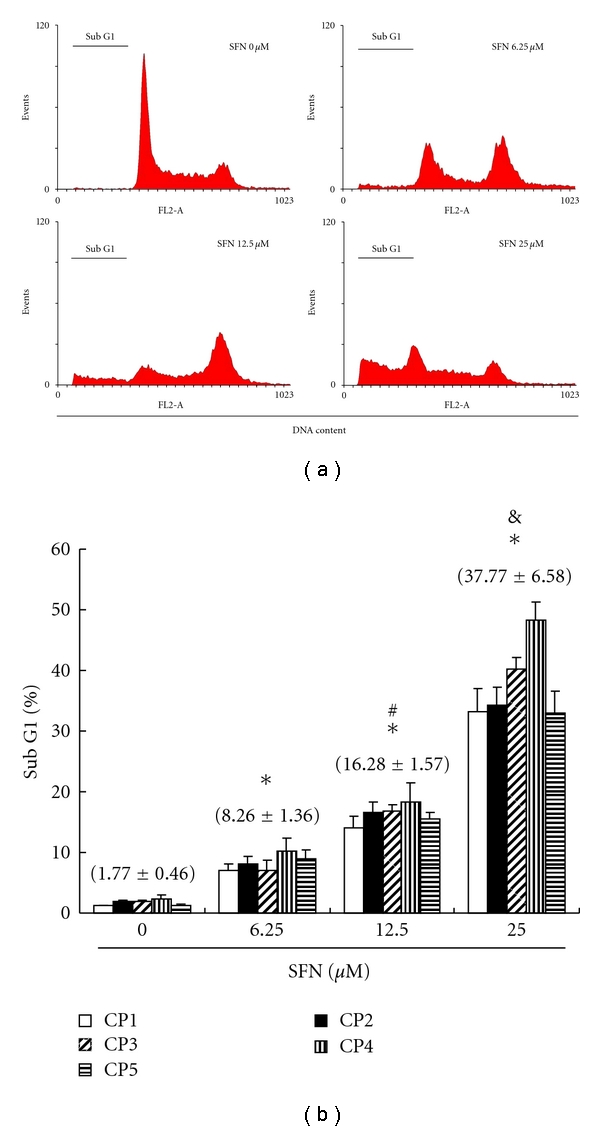
Arrest of cell cycle progression at G2/M and subG0/G1 in response to SFN treatment. (a) SFN-induced G2/M (SFN 6.25 *μ*M) and G0/G1 (SFN 12.5 and 25 *μ*M) arrest in primary CRC cell lines. The distribution of the cell cycle of CRC cell lines was assessed by flow cytometry after staining with propidium iodide (PI). (b) Results were expressed as a percentage of subG0/G1. All data were reported as the means (±SEM) of at least three separate experiments. Statistical analysis used the *t*-test, with the significant differences determined at the level of *P < 0.05 versus SFN 0 *μ*M group, while the symbol on the bar denotes the difference which is statistically significant at *P* < 0.05 as compared to the SFN 6.25 (^#^) or 12.5 *μ*M (^&^).

Cell cycle distribution of SFN-treated CRC cell lines was analyzed by flow cytometry, aiming to determine whether the inhibitory effect was due to cell cycle arrest and apoptosis. Before being processed and analyzed, both kinds of cells were exposed to SFN for a total of 24 h. As shown in Figure 2(a), the CRC cells exposed to SFN 6.25 *μ*M showed G2/M arrest, but SFN 12.5 and 25 *μ*M showed increase in the number of cells in the subG0/G1 phase, as compared with that of the untreated cells. These results revealed that SFN could hold up CRC cell line proliferation via caused cell cycle arrest in the G2/M phase and a significant increase in the proportion of cells in the subG0/G1 phase. The results in Figure 2(b) suggest that the five CRC cell lines have accumulated subG0/G1 phase (*y* = 11.602*x* − 12.985  *R* ^2^ = 0.9139) following the SFN treatment for 24 hours. The observations could imply that the primary colorectal cancer cells have undergone apoptosis.

### 3.5. Apoptosis of CRC Cell Lines Induced by SFN

**Figure 3:**
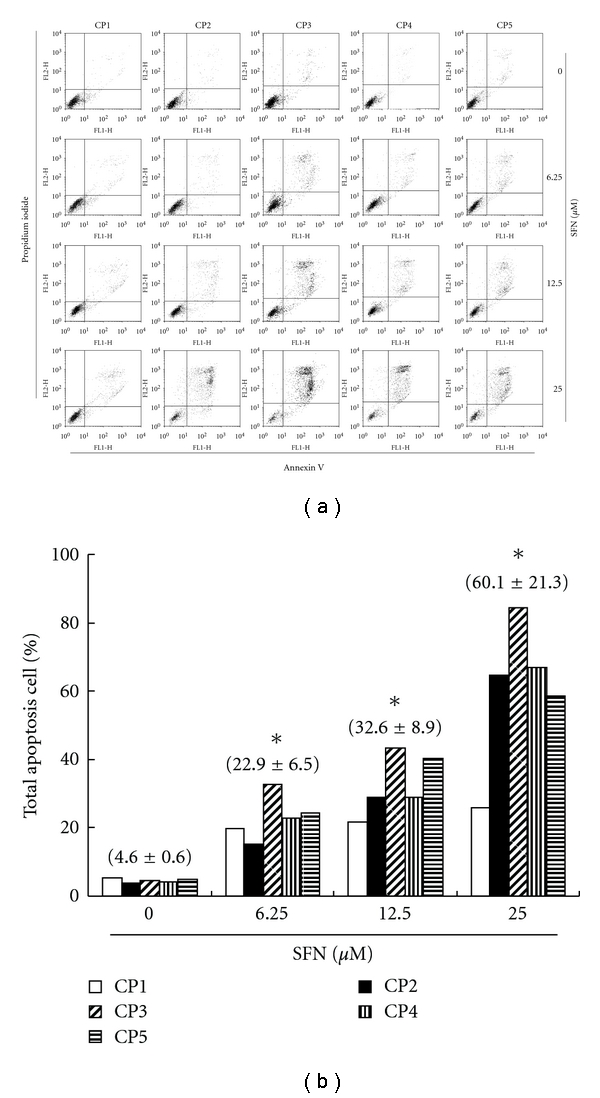
Apoptosis of CRC cell lines induced by SFN. Flow cytometric analysis of PI-Annexin-V to quantify SFN-induced apoptosis in CRC cell lines. (a) Dot plots of five CRC cell lines with SFN treatment at 0, 6.25, 12.5, and 25 *μ*M for 6 h. (b) Results were expressed as a percentage of total apoptosis cells (early and late apoptosis).

Detection between the intact cells, early apoptotic cells, and late apoptotic cells or dead cells could be carried out with PI-annexin-V double staining; thus, we performed this assay to further explore cell apoptosis. To explore the potential role that SFN could play in the apoptosis of CRC cells, the ApopNexin FITC apoptosis detection kit has been used to identify the formation of apoptotic cells in the five CRC cell lines after the 6 hours of exposure to SFN. A typical set of results for the ApopNexin FITC apoptosis detection kit is illustrated in Figure 3(a), in which the annexin V-FITC deposits are indicative of the positive existence of apoptotic cells. A dose-dependent increase in apoptosis was observed (*y* = 17.62*x* − 14  *R* ^2^ = 0.9654), that is, the higher the dose of SFN (6.25, 12.5 and 25 *μ*M) used in the exposure, the greater the extent of apoptosis (Figure 3(b)). The increase of the percentages of apoptotic CRC cell lines was observed in all the doses after treatment for 6 h. In 6 h, approximately 4.6 + 0.6% of five CRC cells were totally apoptotic (early apoptosis and late apoptosis) cells in control. The rate of apoptotic CRC cells increased to 22.9 + 6.5% with 6.25 *μ*M SFN treatments. When the concentrations of SFN increased to 12.5 and 25 *μ*M, the percentages of total apoptotic CRC cells increased to 32.6 + 8.9% and 60.1 + 21.3%, respectively. Taken together, the observations imply that the apoptosis of CRC cell lines is significantly elevated by SFN.

### 3.6. Assessment of Changes in Mitochondrial Membrane Potential

**Figure 4:**
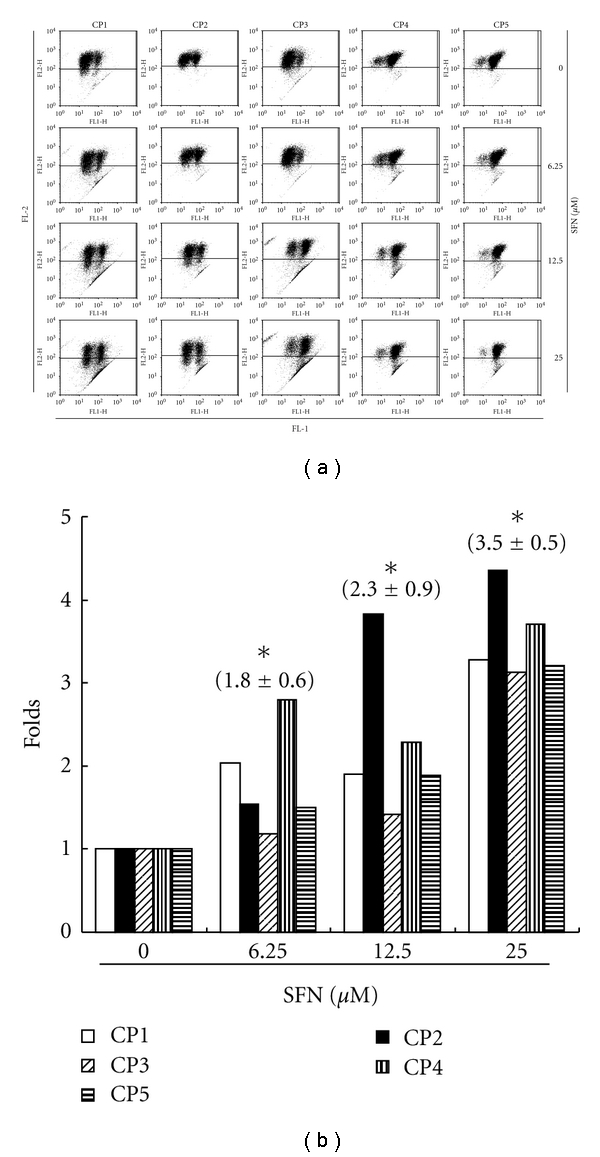
Reduction of the mitochondrial membrane potential (ΔΨ**m**) in the CRC cell lines by SFN, which was determined by JC-1 staining and detected by the flow cytometry. (a) Dot plots of five CRC cell lines with SFN treatment at 0, 6.25, 12.5, and 25 *μ*M for 6 h. (b) The reduction of the ΔΨ**m** containing polarized or depolarized mitochondria determined the ratio of the two fluorescence intensities analyzed by flow cytometry. All the data shown are the folds of five CRC cell lines. The symbol (∗) on each group of bars denotes that difference from the treatment with 0 *μ*M SFN is statistically significant at *P* < 0.05.

The loss of mitochondrial membrane potential is a hallmark for apoptosis. It is an early event coinciding with caspase activation. In nonapoptotic cells, JC-1 exists as a monomer in the cytosol (green) and accumulates as aggregates in the mitochondria, which appear red. In apoptotic and necrotic cells, JC-1 exists in monomeric form and stains the cytosol green.  Figure 4(a) shows typical FL-1/FL-2 dot plots for JC-1 staining CRC cell lines with and without apoptosis. SFN-free CRC cell lines are without apoptosis, which have red fluorescing J-aggregates. The green fluorescing monomers shown in the lower part indicate apoptotic cell lines (SFN 6.25, 12.5, and 25 *μ*M treatment).  Figure 4(b) shows the percentages of apoptotic CRC cell lines analyzed by flow cytometer in different SFN-treated groups. The x-fold increase of mitochondrial membrane potential lost was observed in all the doses after treatment for 6 h. In 6 h, the folds increased to 1.8 + 0.6 with 6.25 *μ*M SFN treatment. When the concentrations of SFN increased to 12.5 and 25 *μ*M, the folds raised to 2.3 + 0.9 and 3.5 + 0.5, respectively. Taken together, the observations imply that SFN has significantly reduced the mitochondrial membrane potential of CRC cell lines.

### 3.7. SFN Inhibits Nuclear NF-*κ*B Transcription Factor Activity in CRC Cell Lines

**Figure 5:**
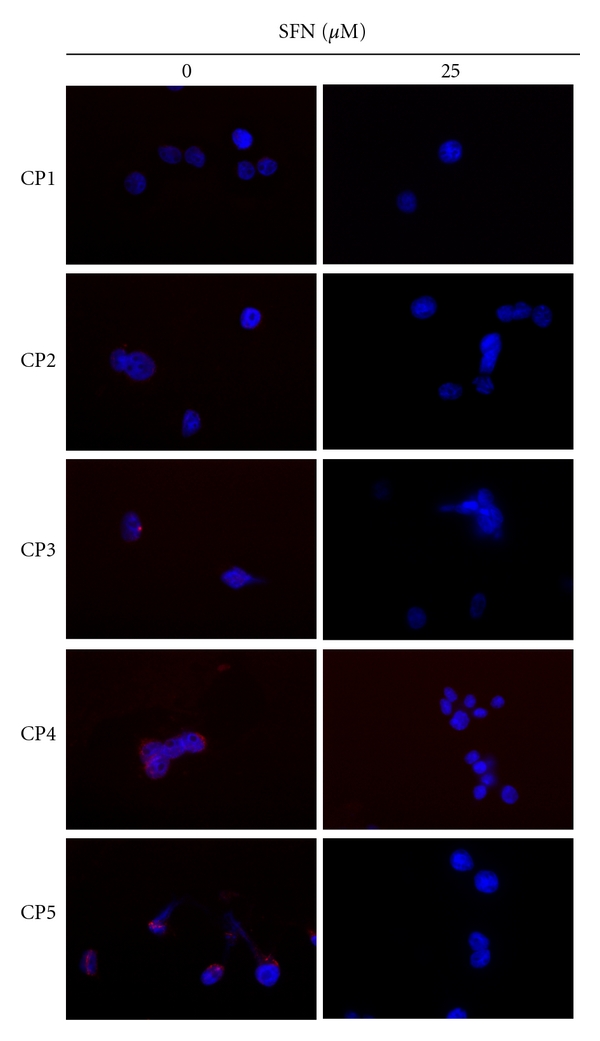
Diminished NF-*κ*B activity in SFN-treated CRC cell lines. Five CRC cell lines were examined for their NF-*κ*B activity 16 hours after SFN stimulation by confocal microscopy of NF-*κ*B subunit p50/52 localization. The CRC cell lines were stained for p50/52 (red). DAPI (blue) indicates nucleus, where active form of NF-*κ*B subunit p50/52 is found.

With finding functional changes and increased apoptosis of SFN in CRC cell lines, the NF-*κ*B activity of SFN-treated CRC cell lines was examined. NF-*κ*B is a transcription factor regulating DC function and apoptosis. NF-*κ*B is inactive in the cytoplasm, as it binds to the protein inhibitor I*κ*B. Following the degradation of I*κ*B, NF-*κ*B p50/52 translocates into the nucleus and binds to its specific target DNA sequence. To explore the potential role that SFN inhibits nuclear NF-*κ*B transcription factor activity of CRC cell lines, confocal microscopy was used to identify the activity of NF-*κ*B transcription factor in the CRC cell lines after the 16 hours of exposure to SFN. The results summarized in Figure 5 indicate that less NF-*κ*B subunit p50/52 was observed in the nuclei of CRC cell lines treated with SFN 25 *μ*M than in the nuclei of SFN-free CRC cell lines. This could imply that the primary colorectal cancer cell lines have repressed activity of NF-*κ*B as per increasing the dose of SFN added into the cell cultures.

### 3.8. Apoptosis Induction by SFN in CRC Cell Lines via Caspase Activation

**Figure 6:**
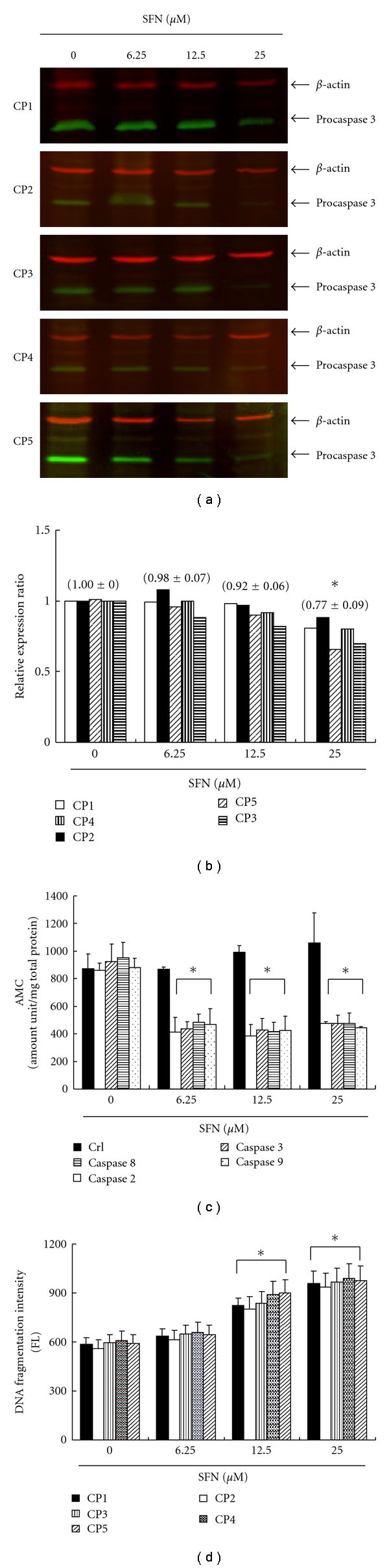
SFN activates procaspase-3 degradation in five CRC cell lines. The cells were treated with SFN (0, 6.25, 12.5, and 25 *μ*M) for 24 hours, and then Western blot analysis was performed for procaspase-3. (a) Representative blot from 3 independent experiments. (b) Quantification of band intensities by Li-COR near infrared imaging system. (c) The caspase-2, -3, -8, -9 activity was analyzed by ApoAlert Caspase assay plates. SFN induces the caspase activity of five CRC cell lines. (d) Quantification of DNA fragmentation by measuring the fluorescence intensities by flow cytometry. The data showed that DNA fragmentation levels were significantly elevated in cells incubated with SFN incubation for 24 hours. All data were reported as the means (±SEM) of five CRC cell lines. Statistical analysis used the *t*-test, with the significant differences determined at the level of *P < 0.05 versus 0 *μ*M control group.

Figure 6(a) shows the immunoblotting of cellular proteins from five CRC cell lines treated with SFN showing decrease of procaspase-3 after SFN incubation. Quantification of procaspase-3, done by measuring the relative band intensities, showed that procaspase-3 levels were significantly lower in cells incubated with SFN (Figure 6(b)). The results indicated that SFN induced caspase-3 activity via cleaved procaspase-3 and apoptosis after SFN incubation. As shown in Figure 6(c), the SFN elevated caspase-2, -3, -8, -9 activities in five CRC cell lines that have been decreased with caspase-specific inhibitors. The results summarized in Figure 6 indicate that the increased levels of caspase activity may play an important role in SFN-induced apoptosis in CRC cell lines.

### 3.9. SFN-Induced DNA Fragmentation in CRC Cell Lines

Cells undergoing apoptosis will lose part of their DNA (due to the DNA fragmentation in later apoptosis) [29]. It is hypothesized that SFN could induce apoptosis of CRC cell lines via DNA fragmentation. To explore this effect of SFN against the five CRC cell lines, an *in vitro* study was initiated by treating each of the cell lines with SFN for 24 hours. The quantification of DNA fragmentation was measured by the fluorescence intensities from flow cytometry (Figure 6(d)), showing that DNA fragmentation levels were significantly increased in cells incubated with SFN. Taken together, the observations imply that SFN has significantly induced the DNA fragmentation of CRC cells.

### 3.10. SFN Inhibits Tumor Growth in SCID Mice

**Figure 7:**
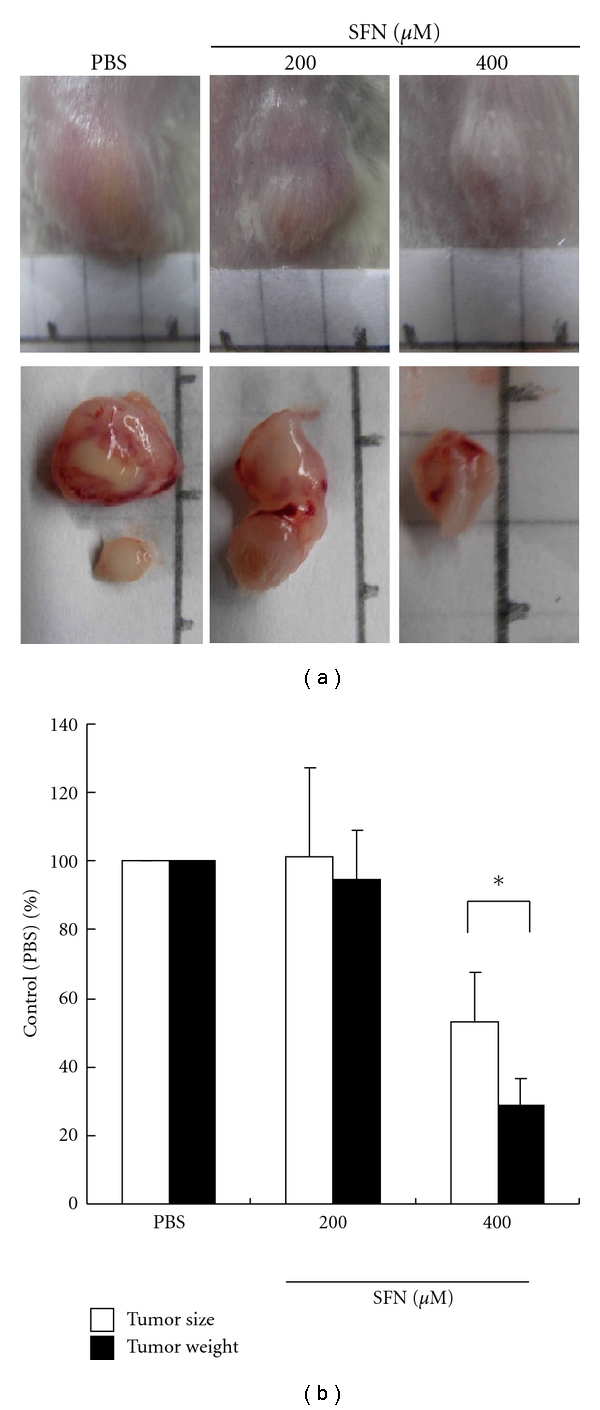
SFN inhibits *in vivo* tumor growth. (a) Effects of SFN on tumor growth of SCID mice subcutaneously inoculated with primary human CRC cell lines. The results showed that SFN 400 *μ*M inhibited tumor growth significantly in both cancer xenografts. (b) Inhibition of tumor growth was compared among tested groups. The inhibitory ratio was normalized to the negative control (PBS group) (values represent percent of control, *n* = 3 *P < 0.05 versus PBS group).

Macroscopic tumors were observed on day 21, after injection of SFN and PBS control groups of nude mice. After injection of SFN 400 *μ*M in the experimental group, tumors developed slowly. After 21 days of injection of SFN, no mice had died in the three groups, and the tumors were resected and measured. Small pale tumor nodules were observed in the tumors, whereas red and hypervascularized large tumors were present in the PBS control and SFN 200 *μ*M tumor cells. The average size of tumors in the experimental group (400 *μ*M) was 52.99% (tumor image size) and 28.70% (tumor weight), much less than the average size of the PBS control (100%) (Figure 7(b)). The results indicated that SFN could decrease tumorigenesis by inhibiting neovascularization in tumors *in vivo*. SFN might also have inhibitory actions on surrounding tumor tissues and indirectly inhibit tumorigenesis (Figure 7(a)).

## 4. Discussion

Sulforaphane (SFN), a naturally occurring alkyl isothiocyanate, has been shown to be a more potent cytotoxic agent than other synthetic analogues isothiocyanates (ITCs) in cancer cells [30]. In a previous study, sulforaphane-treated cells showed growth arrest and cell cycle G2/M accumulation [31]. However, this effect seemed to be independent of a DNA damage Chk1-cdc2-mediated pathway, unlike the G_2_ arrest mediated by radiation, and seemed to be predominantly a metaphase arrest [32]. The effects of SFN may occur by the disruption of microtubules, whereupon it is expected that the activity of the mitotic spindle checkpoint is maintained and arrests cells in metaphase [33]. Of interest, our findings suggest that cell cycle G2/M arrests at the low SFN doses (6.25 *μ*M), whereas apoptosis and caspase activation dominate at the higher doses (12.5 and 25 *μ*M) in five CRC cell lines. The results of mechanical analysis have led us to conclude that both the inhibition of proliferation and the induction of apoptosis are highly dependent upon the SFN. However, on further investigation, our data suggest a more complex mechanism involving cell cycle deregulation and apoptosis that seems to reflect differences in degree of SFN-induced toxicity between the CRC cell lines.

The most common cell death mode on SFN treatment seems to be apoptosis [22]. Two major apoptotic pathways exist: the death receptor and the mitochondrial pathways [34]. Multiple apoptotic stimuli trigger the activation of proteases called caspases, which in turn initiate and execute the apoptotic program [35]. One of the hallmarks of the terminal stages of apoptosis is internucleosomal DNA breakdown, which was first recognized in [36]. Recent studies have led to the discovery of two major apoptotic nucleases, termed DNA fragmentation factor (DFF) [37] or caspase-activated DNase (CAD) [38], and endonuclease G. Both endonucleases attack chromatin to yield 3-hydroxyl and 5-phosphate termini, first creating 50- to 300-kb cleavage products and then oligonucleosomal fragmentation, but these nucleases show different cellular locations and are regulated in fundamentally different ways. Although activation of the executorial caspases seems to be indispensable for realization of the apoptotic program, several forms of cell demise have been shown to be caspase independent or even accelerated by caspase inhibitors [39]. In a previous study, the SFN activated the caspase-8-dependent death receptor pathway, coinciding with the activation of the mitochondrial pathway [40]. Activation of caspase-8 can cleave the BH3 family member Bid [41]. Truncated Bid then migrates to the mitochondria, leading to the loss of MMP, cytochrome *c* release, and activation of the initiator caspase-9 [42]. The observations of this study have implied that SFN has significantly induced the caspase-3 activity of CRC cell lines. Caspase-3-like activity and plasma membrane disintegration served as measures of early apoptosis whereas nuclear fragmentation served as indicator of late apoptosis events [29].

Nuclear factor *κ*B (NF-*κ*B) plays an important role in inflammation, autoimmune response, cell proliferation, and apoptosis by regulating the expression of genes involved in these processes [43]. Active NF-*κ*B is most commonly composed of the heterodimer DNA-binding subunits p50 and p65. It has recently been shown that inactivation of p65 subunit of NF-*κ*B leads to the death through apoptosis of liver cells [44]. Similarly, it has been shown in a wide range of cells that when NF-*κ*B has been inactivated by I*κ*-B*α*, cells were more sensitive to TNF-*α*-induced apoptosis. Evidence exists for NF-*κ*B playing both anti- and proapoptosis roles [45]. The reducing levels of NF-*κ*B may be involved in SFN-induced apoptosis of CRC cells.

Recent studies have shown that SFN inhibited growth of tumor precursors [46] and growth of tumors in mice models when treatment was started at the time of carcinogen administration [47]. The inhibition of PI3K/AKT and ERK pathways acts together to activate FOXO transcription factor and enhances SFN-induced FOXO transcriptional activity, leading to cell cycle arrest and apoptosis [48]. We now show that growth of established s.c. CRC tumor xenografts was suppressed by SFN treatment. Sulforaphane and related isothiocyanate compounds should therefore be investigated further as antitumor agents in addition to their established effects in cancer prevention.

In conclusion, we have demonstrated that SFN inhibits tumor growth by inducing caspase and mitochondrial pathway apoptosis and by arresting the cell cycle at G2/M phase. Our results show that SFN might be an effective anticancer drug for cancer therapy and provide preliminary basis for its clinical application. However, further work is still needed to separate its bioactive components for further drug development.
